# Randomized controlled trial comparing low pressure (8 mmHg) versus high pressure (14 mmHg) CO2 insufflation on postoperative pain in patients undergoing laparoscopic cholecystectomy: Protocol

**DOI:** 10.1371/journal.pone.0339161

**Published:** 2025-12-18

**Authors:** Amine Ben Safta, Salsabil Nasri, Imen Samaali, Hajer Taher, Mohamed Mehdi Trabelsi, Chadli Dziri, Wajih Dougaz, Ramzi Nouira

**Affiliations:** 1 Department of Digestive Surgery B, Charles Nicolle University Hospital, Tunis, Tunisia,; 2 Faculty of Medicine of Tunis, University of Tunis El Manar, Tunis, Tunisia; Government Gousia Hospital, DHS, Srinagar, INDIA

## Abstract

**Background:**

Laparoscopic cholecystectomy is the gold standard for treating symptomatic gallstone disease. Pneumoperitoneum is essential for adequate visualization during the procedure and is typically maintained at a standard pressure of 12–14 mmHg. However, high insufflation pressure may contribute to increased postoperative pain. This study aims to assess whether low-pressure pneumoperitoneum (8 mmHg) reduces postoperative pain compared to standard pressure (14 mmHg), without compromising operative safety or efficacy. We hypothesize that low pressure pneumoperitoneum at 8 mmHg reduces postoperative pain, without increasing operation time or postoperative morbidity.

**Methods:**

This is a single-center, randomized, controlled, double-blind trial. Consecutive Adult patients scheduled for elective laparoscopic cholecystectomy will be randomized in a 1:1 ratio to receive either low-pressure (8 mmHg) or standard-pressure (14 mmHg) pneumoperitoneum. The primary outcome is postoperative abdominal pain assessed using the Visual Analog Scale (VAS) at 6 hours after surgery. Secondary outcomes include VAS pain scores at 12 and 24 hours, postoperative nausea and/or vomiting, postoperative hospital stay and postoperative 30-day morbidity. The enrollment of patients will be done between November 12, 2024 to December 31, 2025.

**Trial registration:**

ClinicalTrials.gov NCT06685250

**Conclusion:**

If proven effective, low-pressure pneumoperitoneum may represent a simple strategy to improve postoperative comfort in laparoscopic cholecystectomy without compromising surgical outcomes.

## Introduction

Laparoscopic cholecystectomy is the gold standard for symptomatic gallstones [1]. It involves the use of Carbone dioxide to inflate the peritoneal cavity to create a pneumoperitoneum in order to provide an adequate visualization and a sufficient space for working. There is no consensus on the optimal pressure of the pneumoperitoneum, but the standard varies between 12 and 14 mmHg [2]. Different studies have demonstrated that high pneumoperitoneum pressure is associated with postoperative morbidity, particularly cardiopulmonary complications and postoperative pain [3]. For this reason, the low impact surgery concept has emerged [4]. Several meta-analyses have compared low pressure versus standard pressure in laparoscopic cholecystectomy and the results were controversial, with high risk of bias across studies [2,5].

### Aim

We aimed to compare postoperative pain (low pressure 8 mmHg versus high pressure 14 mmHg), as a primary outcome, in patients undergoing laparoscopic cholecystectomy for uncomplicated gallbladder lithiasis.

### Hypothesis

The low pressure pneumoperitomenum at 8 mmHg reduces the postoperative pain, at postoperative 6th hour assessment, in patients undergoing laparoscopic cholecystectomy, compared to pneumoperitoneum at 14 mmHg.

## Methods

### Design

This is a single-center prospective randomized controlled trial, double-blinded, parallel-group trial involving patients requiring laparoscopic cholecystectomy for uncomplicated gallbladder lithiasis. The expected start and end dates for this study are November 12, 2024 and December 31, 2025.

This protocol has been developed in accordance with the Standard Protocol Items: Recommendations for Interventional Trials (SPIRIT) guidelines and checklist (attached S1 File) [6]. The study was registered on the ClinicalTrials.gov within the number **NCT06685250 on November 11, 2024** (attached S2 File).

### Study setting

Recruitment will take place at department B of general surgery of Charles Nicolle university hospital of Tunis, Tunisia. This department performs a minimum of six laparoscopic cholecystectomy (LC) per week (for complicated and uncomplicated gallbladder lithiasis).

### Study population

Consecutive patients admitted for laparoscopic cholecystectomy for uncomplicated gallbladder lithiasis will be assessed for eligibility. Screening and enrolment will proceed until the target number of participants is reached.

### Eligibility criteria

- **Inclusion criteria:** (consecutive if possible) adult patients (≥18 years) with uncomplicated symptomatic gallbladder lithiasis scheduled for elective surgery (ASA 1 or ASA 2)
- **NON Inclusion criteria:** Were not included patients with: choledocholithiasis, associated surgical procedures, cerebrovascular accident (with neurological sequelae or other neurological disorders that affect sensation of pain), ascites, carcinomatosis
- **Exclusion criteria: were patients who had** conversion to laparotomy **or** need to keep an Escat’s drain

### Intervention

#### Standard of care.

In all patients, gas pressure was set to 12 mmHg during placement of four ports. Later on, in the high-pressure group the procedure was performed at 14 mm Hg and in the low-pressure group the procedure was performed at 8 mmHg pressure.

Postoperative analgesia will be delivered to all patients respecting the same protocol: 1g of paracetamol/IV which will be administered 30 minutes before the end of the intervention.

#### Control group: standard pneumoperitoneum pressure 14mmHg.

CO2 pneumoperitoneum insufflation at standard pressure (14 mmHg) throughout the procedure

#### Intervention group: low pneumoperitoneum pressure 8 mmHg.

Insufflation of pneumoperitoneum with low-pressure CO2 (8 mmHg) after introduction of standard-pressure trocars

#### Strategies to improve adherence to interventions.

To ensure adherence to the intended stable pneumoperitoneum throughout the intervention, several measures will be implemented. The gas circuit, including the tightness of the insufflation pipe and filters, will be checked before starting the intervention. The healthcare professional administering the intervention, part of the core team of senior physicians and the operating room nurse, will supervise each session.

### Informed consent

Written informed consent will be obtained from all eligible participants. The investigators will explain the study to each potential participant, provide them with an informed consent form, and answer any questions they may have. Written informed consent will be obtained from each participant prior to enrolment (attached S3 File).

### Randomization

Prior to enrolment, all inclusion and exclusion criteria will be thoroughly assessed. Eligible participants will be randomly assigned to one of two parallel groups using **block randomization with a fixed block size of four**, to ensure a progressive balance between arms throughout the inclusion period.

The randomization sequence was generated using a **table of random numbers** extracted from the reference textbook L’essai thérapeutique chez l’homme by Schwartz D et al, [7]. Allocated study subjects were randomly assigned by, using simple 1:1 non-stratified sequence and a block size of 4 [7].

Allocation was implemented sequentially, according to the randomization list, by a team member **not involved in outcome assessment**, in order to maintain **allocation concealment** and minimize the risk of selection bias.

**Fig 1:**
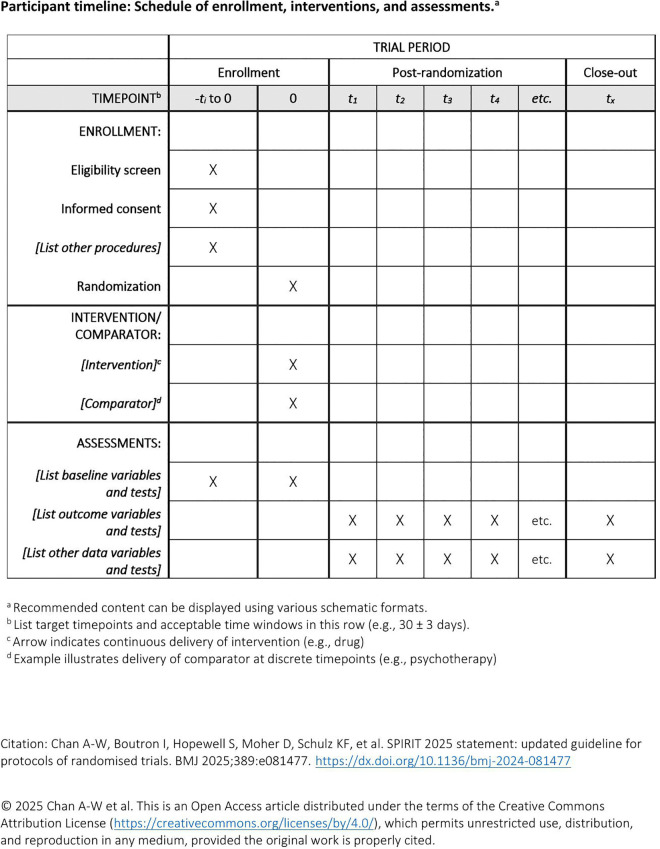
SPIRIT schedule of enrollment.

The timeline for enrolment, intervention and assessments is outlined in the SPIRIT schedule of events in Fig 1

**Fig 2:**
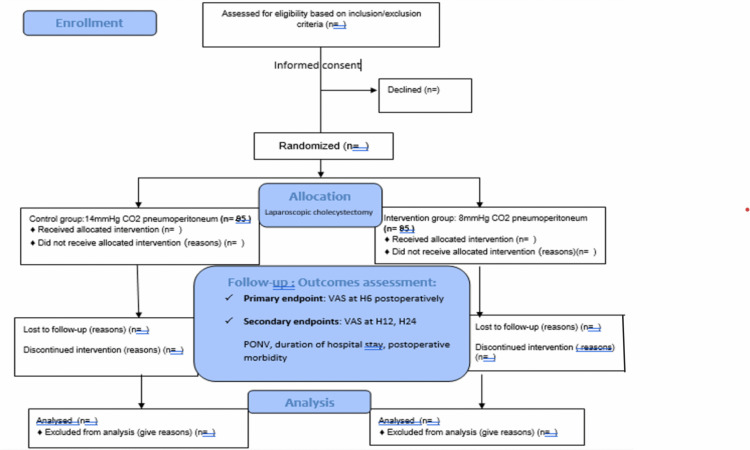
SPIRIT Flow diagram for patients included in the randomized controlled trial comparing postoperative pain after laparoscopic cholecystectomy with standard (14 mmHg) versus low pressure pneumoperitoneum (8mmHh).

The Flowchart of the investigation is illustrated in Fig 2.

### Blinding

The study will follow the principles of the double blinding Randomized Controlled trials [8], which is specifically intended to minimize bias in outcome assessment and data analysis.

To maintain blinding integrity and minimize bias, the participant and the assessor of the postoperative pain were blinded for the allocation. Only the surgeon, the anesthetist and the operating room nurse responsible for opening the allocation envelope and administering the assigned intervention will be aware of the group assignment. These persons will not be involved in outcome assessment, clinical follow-up, or data analysis. The outcome assessor will be a trainee resident. All outcome assessors, trained residents not involved in randomization or intervention delivery, will remain blinded to group allocation throughout the study. Similarly, data analysts will be blinded to treatment assignment to ensure objectivity in statistical analyses and interpretation of results. Anesthetist and surgeon will be explicitly instructed not to disclose their group allocation to any member of the clinical or research team involved in follow-up or outcome assessment. This approach is designed to preserve the integrity of the blinding process and uphold the internal validity of the trial.

### Data collection

#### Baseline assessment (at admission).

Prior to group allocation, each participant’s sociodemographic data (such as age, gender, Body Mass index), baseline characteristics including comorbidities, American Society of Anesthesiologist (ASA) score, preoperative clinical examination, preoperative biological tests, preoperative ultrasound data, and surgical exploration data after trocar placement will be recorded.

Following this assessment, participants will be randomized into one of two study groups.

#### During laparoscopic cholecystectomy.

The presence of eventual adhesions, difficult dissection, bleeding, biliary injury, realization of cholangiography, placement of Escat’s drain, placement of drainage, operation time were recorded.

#### Postoperative assessment.

The postoperative pain at 6th, 12th, 24th hours, the presence of nausea and/or vomiting, the consumption of painkillers, the duration of hospital stay, the presence of eventual complications within 30 days were recorded.

#### Trial implementation and coordination.

The rigorous implementation of this trial relies on strict protocol adherence clearly defined roles and standardized procedures to ensure methodological integrity and internal validity. Prior to enrolment, all department B of general surgery medical and paramedical staff will be trained on the study protocol and data collection methods during dedicated briefing sessions.

The randomization sequence will be generated by (CD) not involved in recruitment, intervention delivery, or outcome assessment. Daily screening and enrolment of eligible participants will be carried out by site investigators. After obtaining written informed consent and confirmation of eligibility, the senior surgeon will perform the allocation by opening sealed, opaque envelopes containing the group allocation, serving as the **sole custodian** of the envelopes. Participants assigned to the intervention group will be operated under 8 mmHg pneumoperitoneum pressure. Control group participants will be operated under pneumoperitoneum pressure (14 mmHg). All patients will receive 1g of intravenous paracetamol 30minutes before the operation’s end. Outcome assessments will be conducted by well-trained residents who are blinded to participants’ group assignments and data analysis. To minimize bias, these residents will not be involved in recruitment or intervention delivery. All assessors will undergo specific training to standardize data collection procedures and reduce inter-rater variability.

#### Intervention fidelity and data quality assurance.

A standardized protocol governs the delivery of the pneumoperitoneum pressure. Laparoscopic cholecystectomy will be performed according to routine clinical care practices. A comprehensive fidelity monitoring plan will be implemented to ensure consistent and reliable delivery of both the intervention and control conditions. This plan addresses key fidelity domains, including adherence, delivery quality, and data accuracy. After each operation, the surgeon will complete a fidelity log documenting operation time, protocol deviations or intraoperative incident. Data quality will be ensured through double data entry and verification processes. This structured fidelity and quality assurance approach is designed to uphold the trial’s internal validity and support accurate interpretation of the intervention’s effects.

### Study endpoints

#### Primary endpoint.

Postoperative pain at 6th postoperative hour (H6): defined as abdominal and/or interscapular pain measured by a Visual Analog Scale (VAS) as following, from 0 to 10: 0 no pain, 5 moderate pain, 10 worst possible pain.

#### Secondary endpoints.

postoperative pain at H12: defined as abdominal and/or interscapular pain measured by a Visual Analog Scale (VAS) as following: 0 no pain, 10 worst possible pain
Postoperative pain at H24: defined as abdominal and/or interscapular pain measured by a Visual Analog Scale (VAS) as following: 0 no pain, 10 worst possible pain
vomiting: Assessed by: Postoperative Nausea and vomiting (PONV) score defined as follow: 0 no PONV, 1 moderate nausea, 2 moderate vomiting, 3 uncontrollable nausea and/or vomiting
Conversion rate to open procedure
Operative time
morbidity: defined by any surgical complications assessed within 30 days postoperatively
postoperative length of stay

All data will be recorded on a dedicated data collection form (attached S4 File). Patient data will then be analyzed in an anonymized SPSS file.

### Statistical methods

#### Sample size and power.

Using the VAS scale, the mean value of abdominal pain after laparoscopic cholecystectomy with a pressure of 14 mm Hg is estimated in the literature at **4.1 + /-1.8** (H6) [9]. We hypothesize that patients in the low-pressure group will experience significantly lower postoperative pain compared to those in the standard-pressure group (14 mm Hg), with no increase in postoperative morbidity. The difference of means (Δ) is estimated to “01”. For a power level of 90%, a significance level of 5% and a two-way comparison between the two arms of the study, 70 patients/group are needed, i.e., 140 in all. Sample size was overestimated by 20% as per loss of follow-up and/or data discrepancies expectation. An overall sample size of 170 subjects will be enrolled in the study, divided into 85 experimental group vs. 85 placebo group [10]. Allocated study subjects were randomly assigned by, using simple 1:1 non-stratified sequence and a block size of 4.

#### Statistical analysis.

All statistical analyses will be conducted in the intention-to-treat population at a two-sided 5% alpha risk. For each group and at each assessment time, categorical variables will be summarized as numbers and percentages, and continuous variables will be expressed as number, mean, and standard deviation (SD). Quantitative variables with skewed distribution will be presented in terms of median and interquartile range (IQR) (25th-75th percentile).

### Primary endpoint

The primary endpoint is the postoperative pain at 06 hours, assessed by VAS will be compared by Student t test or non-parametric test U of Mann and Whitney when appropriate.

### Secondary endpoints

- **Change of VAS over time:** To evaluate the temporal evolution of VAS (at H6, H12 and H24), analysis of variance (ANOVA) with Bonferroni adjustment will be performed. This statistical approach is particularly suited to repeated measures data.
- Nausea and vomiting: Changes from baseline (H0) to the final time point (H4) will be analyzed within groups using paired ANOVA tests. Between-group comparisons of these changes will be performed using independent ANOVA tests.
- Duration of hospital stay: the mean hospital stays in each group (control vs intervention) will be compared using t-tests or non-parametric test U of Mann and Whitney when appropriate.
- Operative time: the mean operative time in each group (control vs intervention) will be compared using t-tests or non-parametric test U of Mann and Whitney when appropriate.
- Conversion rate: the frequency of conversion rate in each group will be compared by chi²test or fisher exact test when appropriate.
- 30-day morbidity: the frequencies of each postoperative complication in each group will be compared by chi²test or fisher exact test when appropriate.

The statistical analysis will be conducted using SPSS version 26. Analyses will be performed by an independent investigator (CD in the authors’ list) blinded to treatment allocation, ensuring unbiased interpretation of the data.

#### Missing data.

The strategy is divided into two main areas: prevention and statistical treatment by imputation:

**1) Prevention**: To minimize the risk of missing data from the design and conduct of the trial, we took in account the following points:
  - Easily measurable endpoints that are unlikely to be missing were chosen. Data collection is performed by a qualified professional (surgery resident).
  - Patients are hospitalized and are unlikely to leave the hospital on the day of the procedure.
  - The tolerable threshold for missing data is 5%. The Sample size of 140 patients, calculated in sample size and power section, was overestimated by 20% as per loss of follow-up and/or data discrepancies expectation. An overall sample size of 170 subjects will be enrolled in the study, divided into 85 experimental group vs. 85 placebo group.
**2) Practical steps for dealing with missing data**: When missing data exists despite prevention efforts, it is essential to manage it in conjunction with intention-to-treat analysis, which is the preferred analysis method for Randomized Trials.

The most common situation is that the absence of data depends on the observed values of other variables, but not on the missing value itself: MAR (missing at random). The solution is then multiple imputation.

[The absence of data that depends on the missing value itself MNAR (missing Not at random) is exceptional in the design of this trial: primary outcome is postoperative abdominal pain assessed using the Visual Analog Scale (VAS) at 6 hours after surgery].

**3)** A **sensitivity analysis** involves repeating the main analysis using different methods of handling missing data to verify whether the conclusions of the trial remain the same.

#### Data analysis framework.

All analyses will follow the intention-to-treat principle. The primary outcome (VAS pain score at 6 hours) will be compared between groups using an independent t-test or a Mann–Whitney U test if non-normal. An adjusted analysis using ANOVA will be performed with age, sex, BMI, ASA class, and operative time as covariates. Repeated measures of pain (6, 12, and 24 hours) will be analyzed using analysis of variance (ANOVA) with Bonferroni adjustment. Categorical variables, including postoperative nausea and vomiting and complications, will be compared using χ² or Fisher’s exact tests. Continuous secondary outcomes will be analyzed using t-tests or appropriate non-parametric methods. Missing data will be addressed using multiple imputation (fully conditional specification with predictive mean matching), with sensitivity analyses including complete-case and per-protocol analyses. All tests will be two-sided with a significance level of 0.05. Statistical analyses will be performed using SPSS version 26.

### Ethics approval

The study protocol was prospectively approved by the ethical committee at Charles Nicolle university hospital of Tunis, Tunisia (approval number FWA 00032748 -IORG0011243) (attached S5 and S6 Files).

A Data Monitoring Committee (DMC) will not be established for this trial because the risk to participants is minimal. Monitoring of adverse events and safety data will be conducted by the research team, in accordance with standard protocols. Any concerns regarding patient safety will be directly reported to the ethics committee.

## Discussion

This trial aims to investigate the role of low pressure pneumoperitoneum in reducing postoperative pain after laparoscopic cholecystectomy. We hypothesize that low pressure pneumoperitoneum at 8 mmHg reduces postoperative pain, without increasing operation time or postoperative morbidity.

If low pressure pneumoperitoneum is shown to effectively improve postoperative pain after LC, it could lead to several positive outcomes. This includes a potential reduction in the consumption of postoperative pain killer and the duration of hospital stay. In fact, one of the most reported causes of failure of ambulatory cholecystectomy is postoperative pain [11]. These improvements could translate into significant cost-savings for healthcare systems.

### Trial status

As of the time of this publication, the study is actively underway in accordance with protocol version. Recruitment of participants started in November 1st, 2024 and is anticipated to be completed by December 2025. The intervention phase and the last follow-up assessments are expected to be concluded by January 2026. The study is progressing according to the planned timeline, and data collection and analysis will commence thereafter to generate meaningful results which will be published.

## Supporting information

S1 Checklist SPIRIT checklist. (DOC)

S2 Protocol Clinical trial protocol. (DOCX)

S3 File Informed consent. (DOCX)

S4 File Data Collection form. (DOCX)

S5 File Ethical approval. (DOCX)

S6 File Clinical trial protocol submitted to ethics committee. (DOCX)
