# Supplementary material for: Randomized controlled trial comparing low pressure (8 mmHg) versus high pressure (14 mmHg) CO2 insufflation on postoperative pain in patients undergoing laparoscopic cholecystectomy: Protocol
Source: PLoS One. 2025 Dec 18;20(12):e0339161. doi: 10.1371/journal.pone.0339161 (PMC12714267; doi:10.1371/journal.pone.0339161)
Supplement: S2 Protocol — (DOCX) [file pone.0339161.s002.docx]

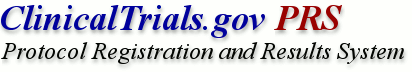


# ClinicalTrials.gov Protocol Registration and Results System (PRS) Receipt

Release Date: November 11, 2024

# ClinicalTrials.gov ID: NCT06685250

**Study Identification**

Unique Protocol ID: Chir-BS-2024-01

Brief Title: Postoperative Pain After a Laparoscopic Cholecystectomy ( CPOP )

Official Title: Randomized Controlled Trial Comparing Low (8 mmHg) Vs. Standard (14 mmHg) CO2 Insufflation Pressure on Postoperative Pain in Patients Undergoing Laparoscopic Cholecystectomy

Secondary IDs:

**Study Status**

Record Verification: November 2024 Overall Status: Not yet recruiting

Study Start: November 12, 2024 [Anticipated]

Primary Completion: November 2, 2025 [Anticipated]

Study Completion: December 2, 2025 [Anticipated]

**Sponsor/Collaborators**

Sponsor: Hopital Charles Nicolle Responsible Party: Principal Investigator

Investigator: Dr Amine BEN SAFTA [ABENSAFTA] Official Title: surgical fellow in General Surgery Affiliation: Hopital Charles Nicolle

Collaborators:

**Oversight**

U.S. FDA-regulated Drug: No

U.S. FDA-regulated Device: No

U.S. FDA IND/IDE: No

Human Subjects Review: Board Status: Approved Data Monitoring:

**Arms and Interventions**

**Study Description**

Brief Summary: Randomized Controlled Trial to compare the effect of low (8 mmHg) versus Standard (14 mmHg) insufflation pressure of CO2 pneumoperitoneum on postoperative pain in patients undergoing laparoscopic cholecystectomy

Detailed Description:

**Conditions**

Conditions: Postoperative Pain Keywords:

**Study Design**

Study Type: Interventional Primary Purpose: Other

Study Phase: N/A

Interventional Study Model: Parallel Assignment Number of Arms: 2

Masking: Double (Participant, Outcomes Assessor) Allocation: Randomized

Enrollment: 190 [Anticipated]

| Arms | Assigned Interventions |
| --- | --- |
| Experimental: Pressure 8 mmHg | Procedure/Surgery: Laparoscopic cholecystectomy  Laparoscopic cholecystectomy |
| Active Comparator: Pressure 14 mmHg | Procedure/Surgery: Laparoscopic cholecystectomy  Laparoscopic cholecystectomy |

**Outcome Measures**

Primary Outcome Measure:

1. Postoperative pain

Visual Analog Scale (VAS) .from 0 to 10 0 no pain, 5 moderate pain, 10 worst possible pain [Time Frame: Hour 6]

Secondary Outcome Measure:

1. postoperative pain

Visual Analog Scale (VAS) 0 no pain, 10 worst possible pain [Time Frame: Hour 12]

1. Postoperative pain

Visual Analog Scale 0 no pain, 10 worst possible pain [Time Frame: Hour 24]

1. vommiting (PONV score)

Postoperative Nausea and vomiting (PONV) score 0 no PONV , 1 moderate nausea, 2 moderate vomiting, 3

uncontrollable nausea and/or vomiting

[Time Frame: from 0 to 24 hours after surgery]

1. morbidity

[Time Frame: 30 days]

1. postoperative length of stay [Time Frame: 30 days]

**Eligibility**

Minimum Age: 18 Years Maximum Age:

Sex: All Gender Based: No

Accepts Healthy Volunteers: No

Criteria: Inclusion Criteria:

- ASA 1 or ASA 2
- uncomplicated symptomatic gallblader lithiasis scheduled for elective surgery
- no choledocholithiasis
- no associated surgical procedures
- no cerebrovasculare accident with neurological sequelae or other neurological disorders that affects sensation of pain
- no ascites
- no carcinomatosis Exclusion Criteria:
- conversion to laparotomy
- need to keep an escat drain

**Contacts/Locations**

Central Contact Person: Amine Ben Safta, Assistant hospitalo-universita

Telephone: +216 29 693 979 Email: [amine.bsa@gmail.com](mailto:amine.bsa@gmail.com)

Central Contact Backup:

Study Officials:

Locations:

**IPDSharing**

Plan to Share IPD:

**References**

Citations:

Links:

Available IPD/Information:

U.S. National Library of Medicine | U.S. National Institutes of Health | U.S. Department of Health & Human Services
