# Supplementary material for: Randomized controlled trial comparing low pressure (8 mmHg) versus high pressure (14 mmHg) CO2 insufflation on postoperative pain in patients undergoing laparoscopic cholecystectomy: Protocol
Source: PLoS One. 2025 Dec 18;20(12):e0339161. doi: 10.1371/journal.pone.0339161 (PMC12714267; doi:10.1371/journal.pone.0339161)
Supplement: S3 File — (DOCX) [file pone.0339161.s003.docx]

**PATIENT INFORMATION AND CONSENT FORM**

1. **Study Title:**

Analgesic effect of low-pressure CO₂ pneumoperitoneum (8 mmHg) during laparoscopic cholecystectomy.

1. **Objective of the Study:**

To evaluate the effect of low-pressure CO₂ pneumoperitoneum (8 mmHg) during laparoscopic cholecystectomy on the reduction of abdominal pain.

1. **Patient Information**

**Name:** .......................................................
**Surname:** .......................................................
**Date of Birth:** .......................................................
**Phone:** .......................................................
**Address:** .......................................................
**Patient study number:** .......................................................

After reviewing the objectives of the study, I declare the following:

I have been clearly and fully informed about the aims, conditions, and stages of the study.
I was granted sufficient time to reflect and decide freely about my participation.
I was given the opportunity to ask all necessary questions to ensure full understanding.

I have been informed that my decision to participate or not in this study is entirely voluntary, and that I may withdraw from the study at any time, simply by notifying the investigator, without any prejudice to the quality of care or medical services I receive.

I acknowledge that this study has obtained the approval of the Medical Ethics Committee on the date of ................................, in accordance with applicable laws and regulations.

I understand that this research does not impose any particular treatment or medication change on me or my attending physician unless I choose to do so.

I have been informed that my personal information may be used for research purposes, in a strictly confidential manner, and will only be accessed by authorized individuals under the supervision of the principal investigator.

I agree that the principal investigator, or their delegate, may handle the data collected in this study in an anonymous and confidential manner.

I have been informed that, upon completion of this study, I will be informed of the overall results by my attending physician once they become available.

I understand that my consent does not release the investigators or the official sponsor of the study from any of their legal or ethical responsibilities, and that I retain all my rights under the law.

1. **For the Investigator**

**Investigator’s name:** .......................................................
**Phone number:** .......................................................
**Date:** .......................................................
**Signature:** .......................................................

1. **For the Patient**

I freely agree to participate in this study under the terms described above, after reading and approving them.

**Date:** .......................................................
**Signature:** .......................................................

1. **For the Witness**

I, the undersigned ....................................................... (Name and Surname), hereby declare that I have no relation to the investigator or the study sponsor.
I certify that ....................................................... (Name and Surname of the patient) has clearly and explicitly expressed their consent to participate in the study.

**Date:** .......................................................
**Signature:** .......................................................

**If the patient is unable to sign, please specify the reason:**
..................................................................................................................
..................................................................................................................

ـــــــــــــــــــــــــــــــــــــــــــــــــــــــــــــــــــــــــــــــــــــــــــــــــــــــــــــــــــــــــــــــــــــــــــــــــــــــــــــــــــــــــــــــــــــــــــــــــــــــ

عنوان الدراسة:

**Objectif de l’étude :**

Effet antalgique d’un pneumopéritoine au CO2 à basse pression (8 mm Hg) au cours d’une cholécystectomie laparoscopique.

**أهداف الدراسة:**تقييم تأثير الضغط المنخفض لغاز ثاني أكسيد الكربون 8) مم زئبقي)، أثناء إجراء عملية استئصال المرارة بواسطة المنظار في تخفيف ٱلام البطن.

خاص بالمريض

| الاسم....................................................................... اللقب :......................................................  تاريخ الميلاد:.......................................... الهاتف:....................................................  العنوان:..............................................................................رقم المريض في الدراسة :........................ |
| --- |

بعد الاطلاع على اهداف الدراسة اصرح:

1. بانني اخبرت بكل جلاء ووضوح عن اهداف وظروف و اطوار الدراسة ، وانني امهلت فترة كافية للتدبر و التفكير قبل الادلاء بقرار الموافقة على المشاركة في هاته الدراسة وانني قد منحت فرصة بسط كل الأسئلة الضرورية والمكملة لمعلوماتي
2. بانني اخبرت بان قراري في المشاركة او عدم المشاركة في هذه الدراسة امر راجع لي كما انه بإمكاني الانسحاب من هذه الدراسة في أي وق ت ارغب فيه في ذلك مع ابلاغ الطبيب المنظم لهذا البحث ،بدون أي ضير ولا اذية لجودة العناية والخدمات الصحية المقدمة لي
3. بانني لاحظت و ان الدراسة قد حصلت على الموافقة من لجنة الاخلاقيات الطبية بتاريخ ........................ وبمقتضى القوانين الجارية المفعول.
4. بان هذا البحث لا يملي علي ولا على الطبيب المباشر على معالجتي، طريقة ولا نوعية الدواء الذي اتعاطاه، ولا يلزمني بتغييره اذا لم ارغب في ذلك عند الاقتضاء.
5. بانني اخبرت انه من الممكن ان تكون المعلومات الشخصية موضوع بحث ودراس ات بطريقة تحفظ سريتها، وضمانها من طرف اشخاص معينين من الطبيب المسؤول على هذه الدراسة
6. انني اقبل بان يقوم الطبيب المسؤول على هذه الدراسة او من ينوبه بمعالجة معطيات البيانات التي تسجل في هذه الدراسة بصفة خفية
7. انني أبلغت بانه في نهاية هذا البحث سوف أكون على اطلاع على النتائج الشاملة للدراسة بواسطة طبيبي المعالج،الذي سيخبرني بها عندما تكون جاهزة

ان موافقتي لا تخلي مسؤولية منظمي الدراسة و الراعي الرسمي لها باي حال من الأحوال،وانني احتفظ بجميع حقوقي المنصوص عليها بالقانون.

ــــــــــــــــــــــــــــــــــــــــــــــــــــــــــــــــــــــــــــــــــــــــــــــــــــــــــــــــــــــــــــــــــــــــــــــــــــــــــــــــــــــــــــــــــــــــــــــ

| **خاص بالطبيب الباحث** | **خاص بالمريض** |
| --- | --- |
| **لقب الباحث:**  **رقم الهاتف:**  **التاريخ:**  **التوقيع**: | انني أقبل بمحض ارادتي المشاركة في هذه الدراسة وبمقتضى الشروط الوارد ذكرها آنفا :أورد العبارة "بعد الاطلاع والموافقة "  **التاريخ:**  **التوقيع:** |

| **خاص بالشاهد** |
| --- |
| اني الممضي اسفله ..................................(اسم ولقب الشاهد) أشهد انه لا تربطني أي علاقة بالطبيب الباحث ولا مع الراعي الرسمي لهاته الدراسة وان ................................(اسم ولقب المريض) قد اعرب(ت) بكل وضوح وجلاء عن قبوله المشاركة في الدراسة  التاريخ: ...............................................................التوقيع:.................................................  دوافع عدم استجابة المريض على التوقيع :...................................................................................  .................................................................................................................................. |
