# Supplementary material for: Randomized controlled trial comparing low pressure (8 mmHg) versus high pressure (14 mmHg) CO2 insufflation on postoperative pain in patients undergoing laparoscopic cholecystectomy: Protocol
Source: PLoS One. 2025 Dec 18;20(12):e0339161. doi: 10.1371/journal.pone.0339161 (PMC12714267; doi:10.1371/journal.pone.0339161)
Supplement: S4 File — (DOCX) [file pone.0339161.s004.docx]

**DATA COLLECTION FORM**

**Patient ID number:
Patient Full name:
Hospital record number:
Phone number:**
**Date of birth:**
**Address:**

| Male or female over 18 years of age. |  |
| --- | --- |
| Able to understand the objectives of the study and having signed informed consent. |  |
| ASA Physical Status I or II |  |
| Symptomatic uncomplicated gallbladder stones scheduled for elective laparoscopic cholecystectomy |  |
| No common bile duct stones (no choledocholithiasis). |  |
| No associated surgical procedure planned. |  |
| No history of stroke with neurological sequelae or other sensory deficits |  |
| No ascites. |  |
| No peritoneal carcinomatosis. |  |

1. **Eligibility Criteria:**
2. **Eligibility:**

| The patient meets the inclusion criteria. |  |
| --- | --- |
| Date of inclusion |  |

1. **Study Treatment:**

| Date of surgery |  |
| --- | --- |
| Date of randomization envelope opening |  |
| The standard general analgesia protocol was properly conducted:   - Intravenous paracetamol administered 30 minutes before the end of surgery. - Intramuscular tramadol 100 mg administered 30 minutes before the end of surgery. |  |

1. **Sociodemographic Characteristics:**

| Age |  |
| --- | --- |
| Sex |  |
| Weight (kg) |  |
| Height (cm) |  |
| Body Mass Index (BMI) (kg/m²) |  |

1. **Medical History / Comorbidities:**

| Smoking history (pack-years) |  |
| --- | --- |
| Alcohol consumption |  |
| Hypertension |  |
| Diabetes mellitus |  |
| Cardiovascular or peripheral arterial disease |  |
| Chronic respiratory disease |  |
| Chronic kidney disease |  |
| Liver cirrhosis / Portal hypertension |  |
| Corticosteroid or immunosuppressive therapy |  |
| Obesity |  |
| Prior abdominal surgery (open/laparoscopic) |  |
| ASA physical status |  |

1. **Clinical Presentation:**
2. **Symptom:**

| **Symptom** | **Present (Yes/No)** | **Duration** |
| --- | --- | --- |
| Biliary colic |  |  |
| Recent worsening of pain |  |  |
| Epigastric pain |  |  |
| Right upper quadrant pain |  |  |
| Vomiting |  |  |
| Fever |  |  |
| Jaundice |  |  |

1. **Physical Examination:**

| **Physical Examination** | **Finding** | **Observation** |
| --- | --- | --- |
| Fever |  |  |
| Jaundice |  |  |
| Abdomen soft |  |  |
| Abdominal tenderness |  |  |
| Guarding |  |  |
| Rigidity |  |  |
| Previous abdominal scar |  |  |

1. **Laboratory Tests:**

| Blood group |  |
| --- | --- |
| Hemoglobin (g/dL) |  |
| Total bilirubin (µmol/L) |  |
| Conjugated bilirubin (µmol/L) |  |
| GGT (U/L) |  |
| ALP (U/L) |  |
| AST (U/L) |  |
| ALT (U/L) |  |

1. **Abdominal Ultrasound Findings**

| Maximal transverse diameter (cm) |  |
| --- | --- |
| Gallbladder wall thickness (mm) |  |
| Macrolithiasis |  |
| Microlithiasis |  |
| Impacted stone |  |
| Pericholecystic fluid |  |
| Intraperitoneal fluid |  |

1. **Intraoperative Data**

| Surgeon |  |
| --- | --- |
| Date |  |
| Delay from first consultation (days) |  |

1. **Operative Exploration**

| Number of ports |  |
| --- | --- |
| Trocar insertion under standard pressure (12 mmHg) |  |
| Adhesions (none / mild / severe) |  |
| Pericholecystic fluid |  |
| Gallbladder not distended |  |
| Thin-walled gallbladder |  |
| Cystic duct of normal caliber |  |
| No inflammatory changes around the cystic pedicle |  |

1. **Randomization**

| Yes → Group (A / B) : |  |
| --- | --- |
| No → Reason : |  |

1. **Operative Procedure**

| Calot’s-first (infundibulum-first) |  |
| --- | --- |
| Fundus-first (top-down) |  |
| Placement of a Redon drain |  |
| Intraoperative gallbladder perforation |  |
| Peritoneal lavage |  |
| Duration of surgery (minutes) |  |
| Intraoperative cholangiography |  |
| Transcystic drain left in place |  |
| Failure to maintain low pressure → conversion to standard pressure |  |
| Conversion to open surgery (laparotomy) |  |
| Associated surgical procedure |  |
| Analgesia at the end of surgery (as per protocol) |  |

1. **SUITES OPERATOIRES**

| Length of hospital stay (days) |  |
| --- | --- |
| Uncomplicated course |  |
| Postoperative complications |  |

1. **Type de complication**

| Thromboembolic event |  |
| --- | --- |
| Pneumonia |  |
| Urinary tract infection |  |
| Postoperative peritonitis |  |
| Ileus / delayed bowel movement |  |
| Abdominal wall abscess |  |
| Wound dehiscence |  |
| Intra-abdominal collection |  |
| Postoperative bowel obstruction |  |
| Biliary fistula |  |
| Death |  |

1. **Postoperative Vomiting Assessment**

| Presence of vomiting |  |
| --- | --- |
| Time after surgery (hours) |  |
| Frequency (episodes/24 h) |  |

1. **Pain Assessment**

| **Time** | **VAS score** | **Location** | **Observation** |
| --- | --- | --- | --- |
| **H6** |  |  |  |
| **H12** |  |  |  |
| **H24** |  |  |  |

1. **Analgesic Consumption**

| **Analgesic** | **Dose** | **Time after surgery** | **Frequency (per 24 h)** | **Route of administration** |
| --- | --- | --- | --- | --- |
| Paracetamol |  |  |  |  |
| Tramadol |  |  |  |  |
| Acupan (nefopam) |  |  |  |  |
| Weak opioid analgesic (oral) |  |  |  |  |
| Morphine |  |  |  |  |
| Other (specify) |  |  |  |  |
