# Supplementary material for: Randomized controlled trial comparing low pressure (8 mmHg) versus high pressure (14 mmHg) CO2 insufflation on postoperative pain in patients undergoing laparoscopic cholecystectomy: Protocol
Source: PLoS One. 2025 Dec 18;20(12):e0339161. doi: 10.1371/journal.pone.0339161 (PMC12714267; doi:10.1371/journal.pone.0339161)
Supplement: S5 File — (DOCX) [file pone.0339161.s005.docx]

**Randomized Controlled Trial Comparing Low pressure (8 mmHg) versus High pressure (14 mmHg) CO2 Insufflation on Postoperative Pain in Patients Undergoing Laparoscopic Cholecystectomy: Protocol**

**Study title**

Randomized Controlled Trial Comparing Low pressure (8 mmHg) versus High pressure (14 mmHg) CO2 Insufflation on Postoperative Pain in Patients Undergoing Laparoscopic Cholecystectomy: Protocol

**NTC Code.** NCT06685250

**Principal investigator**

Dr. BEN SAFTA AMINE, Assistant in General Surgery, Surgery Department B, Charles Nicolle Hospital, Tunis

**Co-Investigators**

Dr. Nasri Salsabil, Assistant in General Surgery, Surgery Department B, Charles Nicolle Hospital, Tunis

Dr. Imen Samaali, Associate Professor in General Surgery, Surgery Department B, Charles Nicolle Hospital, Tunis

Dr. Hajer Tahar, Resident in General Surgery, Surgery Department B, Charles Nicolle Hospital, Tunis

Dr. Trabelsi Mehdi, Assistant in General Surgery, Surgery Department B, Charles Nicolle Hospital, Tunis

**Nature of the study.** Phase III

**Background**

Laparoscopic cholecystectomy is the gold standard for treating symptomatic gallstone disease. Pneumoperitoneum is essential for adequate visualization during the procedure and is typically maintained at a standard pressure of 12–14 mmHg. However, high insufflation pressure may contribute to increased postoperative pain. This study aims to assess whether low-pressure pneumoperitoneum (8 mmHg) reduces postoperative pain compared to standard pressure (14 mmHg), without compromising operative safety or efficacy.

We hypothesize that low pressure pneumoperitoneum at 8mmHg reduces postoperative pain, without increasing operation time or postoperative morbidity.

**Experimental design**

Randomized comparative study with two parallel groups: “low-pressure CO₂ pneumoperitoneum at 8 mmHg during laparoscopic cholecystectomy” versus “standard-pressure CO₂ pneumoperitoneum at 14 mmHg”.

**Aim**

Primary: Assess the effectiveness of low-pressure CO₂ pneumoperitoneum during laparoscopic cholecystectomy in reducing postoperative pain.

**Study Population**

Patients presenting with isolated, uncomplicated symptomatic gallstones who undergo laparoscopic cholecystectomy in Surgery Department B, Charles Nicolle Hospital, Tunis.

- Inclusion criteria:

Patient:

- Male or female > 18 years

- ASA I or II

- Informed consent obtained

Disease:

- Symptomatic gallstones

- Scheduled laparoscopic cholecystectomy

- Non-inclusion criteria (preoperative):

Patient:

- < 18 years old

- Refusal to consent

- ASA III or IV

Disease:

- Complicated gallstones (cholangitis, cholecystitis, pancreatitis)

- Associated common bile duct stones

- Pathology associated with gallstones

**Study treatments**

According to randomization, patients will undergo:

- Insufflation of low-pressure CO₂ pneumoperitoneum (8 mmHg) after trocar insertion at standard pressure, or

- Insufflation of standard-pressure CO₂ pneumoperitoneum (14 mmHg) throughout surgery

**Data collected:**

- Selection criteria

- Sociodemographic data

- Preoperative clinical parameters

- Preoperative laboratory parameters

- Intraoperative findings

- Study treatment allocation

- Clinical follow‑up and postoperative investigations

**Outcome measures**

- Primary outcome:

- Abdominal and interscapular pain at 6 hours postoperatively, assessed by VAS (Visual Analog Scale)

- Secondary outcomes:

- Abdominal and interscapular pain at 12 h and 24 h post-op

- Postoperative vomiting within 24 h

- Postoperative morbidity up to 30 days

- Length of postoperative hospital stay

**Follow‑up schedule**

- Inclusion visit verifying eligibility

- Hospital period with evaluations of pain at H6, H12, and H24

- Outpatient follow‑up visit between Day 10 and Day 15

**Study duration**

- Inclusion period: 12 months

- Follow‑up: 1 month

**Randomization**

Performed by Dr. Dziri. Eligible participants will be randomly assigned to one of two parallel groups using **block randomization with a fixed block size of four**, to ensure a progressive balance between arms throughout the inclusion period.

The randomization sequence was generated using a **table of random numbers** extracted from the reference textbook L’essai thérapeutique chez l’homme by Schwartz D et al, [1]. Allocated study subjects were randomly assigned by, using simple 1:1 non-stratified sequence and a block size of 4 [1].

**Primary outcome assessment**

Pain at H6 will be assessed systematically using the VAS scale in a double‑blind manner. The assessor will not know the patient’s group.

**Protocol violation**

Defined for any selected patient with:

- Randomization allocation error

- Pain assessment performed outside the 6‑hour postoperative window

**Time windows**

- Inclusion date = day patient fulfills all inclusion and exclusion criteria

- Surgery date = day of procedure

- Study start date = date the randomization envelope is opened

- H6 postoperative pain assessment = 6 h after end of surgery

**Sample size**

Using VAS scores, the average postoperative abdominal pain after laparoscopic cholecystectomy is estimated at 4,1 ± 1.8 at H6 [2]. For 90% power, alpha = 5%, and bilateral comparison, 70 patients per group are required (total 140) [3]. With 10% attrition, 170 patients are required: 85 in the experimental group and 85 in the control group.

**Statistical analysis plan**

- Definition of analysis populations and baseline characteristics

- Comparison of the two groups

- Analysis of primary outcome

- Analysis of secondary outcomes

**Ethics**

The study will begin after approval from the Ethics Committee of Charles Nicolle Hospital.

**Communication and publication**

Results will be published in a surgical journal.

**References**

1. Schwartz D, Flamant R, Lellouch J. *L’essai thérapeutique chez l’homme*. Paris: Flammarion; 1970. 297 p.

2. Koc M, Ertan T, Tez M, Kocpinar MA, Kilic M, Gocmen E, et al. Randomized, prospective comparison of postoperative pain in low- versus high-pressure pneumoperitoneum. ANZ J Surg. août 2005;75(8):693‑6

3. Schwartz D, Flamant R, Lelouch J. *L’essai thérapeutique chez l’homme*. Paris: Flammarion; 1970. 278 p (table 6), 282 p (table 10b).
